# Structural characterisation of the catalytic domain of botulinum neurotoxin X - high activity and unique substrate specificity

**DOI:** 10.1038/s41598-018-22842-4

**Published:** 2018-03-14

**Authors:** Geoffrey Masuyer, Sicai Zhang, Sulyman Barkho, Yi Shen, Linda Henriksson, Sara Košenina, Min Dong, Pål Stenmark

**Affiliations:** 1 Department of Biochemistry and Biophysics, Stockholm University, SE-106 91, Stockholm, Sweden; 2 Department of Urology, Boston Children’s Hospital, Department of Microbiology and Immunobiology and Department of Surgery, Harvard Medical School, Boston, MA 02115 USA

## Abstract

Botulinum neurotoxins (BoNTs) are among the most potent toxins known and are also used to treat an increasing number of medical disorders. There are seven well-established serotypes (BoNT/A-G), which all act as zinc-dependent endopeptidases targeting specific members of the SNARE proteins required for synaptic vesicle exocytosis in neurons. A new toxin serotype, BoNT/X, was recently identified. It cleaves not only the canonical targets, vesicle associated membrane proteins (VAMP) 1/2/3 at a unique site, but also has the unique ability to cleave VAMP4/5 and Ykt6. Here we report the 1.35 Å X-ray crystal structure of the light chain of BoNT/X (LC/X). LC/X shares the core fold common to all other BoNTs, demonstrating that LC/X is a bona fide member of BoNT-LCs. We found that access to the catalytic pocket of LC/X is more restricted, and the regions lining the catalytic pocket are not conserved compared to other BoNTs. Kinetic studies revealed that LC/X cleaves VAMP1 with a ten times higher efficiency than BoNT/B and the tetanus neurotoxin. The structural information provides a molecular basis to understand the convergence/divergence between BoNT/X and other BoNTs, to develop effective LC inhibitors, and to engineer new scientific tools and therapeutic toxins targeting distinct SNARE proteins in cells.

## Introduction

The clostridial neurotoxins are a family of bacterial toxins including seven BoNTs and the related tetanus neurotoxin (TeNT). They are the causative agents of the severe paralytic diseases, botulism and tetanus, respectively^1^. BoNTs are the most poisonous protein toxins known to man, and yet have also been successfully used clinically to treat an ever-increasing number of disorders, such as strabismus, blepharospasm, cervical dystonia, overactive bladder, and pain^2^.

BoNTs consist of a light chain (LC, ~50 kDa) and a heavy chain (HC, ~100 kDa) linked by an inter-chain disulphide bridge^3^. LC is a zinc-dependent endopeptidase, while HC is composed of two functional domains that are responsible for translocation (H_N_) and receptor binding (H_C_)^4^. The toxin acts by first recognizing specific receptors at motor nerve terminals and enters neurons via receptor-mediated endocytosis. The acidic pH in endosomes then causes a conformational change of the toxin, resulting in translocation of LC across the endosomal membrane^3^. LC targets one of three members of the SNARE family. BoNT/A, /C and /E cleave the peripheral membrane protein SNAP-25. BoNT/B, /D, /F, and /G cleave the vesicle-associated membrane protein VAMP1/2/3. BoNT/C can also cleave syntaxin 1^4^. Cleavage of these SNARE proteins blocks fusion of the synaptic vesicles to the plasma membrane and thus inhibits neurotransmission release and causes paralysis.

BoNT-LCs are remarkably specific proteases and the three sets of neuronal SNARE proteins are the only known targets. Furthermore, each BoNT has its own unique cleavage site on their substrates. This specificity is due to extensive toxin-substrate interactions between regions (designated exosites) outside the conserved catalytic site in LCs and regions in the substrates beyond the cleavage site. In particular, a conserved motif in SNARE proteins known as the SNARE motif has been proposed to play crucial roles for recognition by toxins^5^. For instance, VAMP2 contains two copies of the SNARE motif (V1 and V2) and V1 is critical for efficient cleavage of VAMP2 by BoNT/D^7^. Indeed, the co-crystal structure of LC/A in complex with SNAP-25 has defined two exosites (α- and β-) that interact with specific regions in SNAP-25^7^, and the crystal structure of LC/F in complex with peptides derived from VAMP2 also revealed three exosites that interact with VAMP2^8^.

The crystal structures of all BoNT-LCs have been resolved^9–16^. Despite the low degree of identity at the amino acid levels, all LCs display a highly conserved fold, presenting a compact globular aspect with mixed secondary structures of α-helices and β-strands. The catalytic pocket, which contains the HExxE zinc-dependent protease motif, showed similar composition and geometries across all BoNTs. It is likely that variations in the composition and placement of exosites determine which SNARE proteins can be cleaved, as well as the spatial location of the cleavage site on a SNARE protein. In addition, it has been shown that the residue located at the C-terminal side of the cleavage site (P1’ position) in SNARE proteins is critical for efficient cleavage, and mutations at this site usually abolishes the cleavage by BoNTs. For instance, although BoNT/C cleaves both SNAP-25 and syntaxin 1, the P1’ position in these two different substrates are conserved (Ala).

A new serotype of BoNT with unique substrate specificity was recently identified from the genome sequence of *C*. *botulinum* strain 111, and designated BoNT/X^17^. BoNT/X cleaves VAMP1/2/3 between Arg66-Ala67 in VAMP2, which is distinct from the sites targeted by other BoNTs^17^. Remarkably, BoNT/X is the first and only BoNT member that can cleave additional VAMP family proteins, VAMP4, VAMP5 and Ykt6. The cleavage site on VAMP4, VAMP5, and Ykt6 are at the homologous location as Arg66-Ala67 in VAMP2, but the residues at the cleavage site are different (Lys-Ser in VAMP4 and Ykt6, Arg-Ser in VAMP5). Thus, LC/X showed the unique capability to recognize a broad range of VAMPs and a remarkable degree of tolerance for residue changes at the cleavage site.

To understand the structural basis of LC/X action, here we resolved the high resolution X-ray crystal structure of LC/X and of its zinc-free, apo-form. LC/X presents common features with all other clostridial neurotoxins. However the detailed structural characterisation also highlights unique features that may confer LC/X with its unusual substrate specificity and enhanced cleavage efficiency.

## Results and Discussion

### Crystal structure of LC/X

LC/X (residues 1–439) was recombinantly produced in *E*. *coli*, with an N-terminal poly-His(x6) tag. Two crystal forms were obtained under distinct crystallization conditions. In the first form, data from a single crystal were collected up to 1.35 Å resolution (Table 1) and the structure of LC/X was solved using molecular replacement with LC/B as a template. Residues 1–413 were clearly defined, however no electron density was observed for the N-terminal tag and the C-terminal end (414–439), all in solvent-accessible areas. In the second crystal form, the metal chelator EDTA was included in the crystallisation condition, which resulted in the loss of the Zn^2+^ ion from LC/X. Data from a single plate-like crystal were collected up to 2.4 Å resolution and comprises residues 1–423 (Table 1).

**Table 1:** Crystallographic statistics of the LC/X X-ray crystal structures.

	LC/X	Apo-LC/X
PDB code	6F47	6F4E
Synchrotron source, λ	Diamond I02, 0.979 Å	SLS PXI, 1.00 Å
Resolution (Å)	1.35 (1.37–1.35)	2.4 (2.49–2.40)
Space group	*P2*_1_2_1_2_1_	*P22*_1_2_1_
Cell dimensions and angles	a = 58.3, b = 86.7, c = 93.1 Å; α = β = γ = 90°	a = 52.3, b = 94.5, c = 113.0 Å; α = β = γ = 90°
Total/Unique reflections	592,965 98,954	176,299 21,016
Completeness (%)^a^	94.9 (64.8)	93.4 (88.9)
*R*_merge_^a,b^	0.083 (0.98)	0.123 (0.97)
*R*_pim_^a,c^	0.053 (0.74)	0.062 (0.52)
I/σ(I)^a^	9.2 (1.0)	8.9 (2.1)
CC_1/2_^d^	0.99 (0.42)	0.99 (0.69)
Multiplicity	6.0 (3.9)	8.4 (7.8)
*R*_*cryst*_^*e*^ (%)	14.3	21.5
*R*_free_^f^ (%)	17.6	26.0
Rmsd in bond lengths (Å)	0.009	0.009
Rmsd in bond angles (°)	1.34	1.31
**B- factor statistics (Å** ^**2**^ **)**		
Protein all atoms	20.3	53.6
Protein main chain atoms	18.4	51.4
Protein side chain atoms	22.2	55.7
Solvent atoms	36.0	46.8
Zn^2+^ ion	18.2 (0.33 occupancy)	n/a
**Ramachandran statistics (Molprobity)**		
Favored	97.7%	97.1%
Outliers	0.0%	0.0%

^a^Values in parentheses refer to the highest resolution shell.

^b^*R*_merge_ = ΣΣ_*i*_|*I*_*h*_ − *I*_*hi*_|/ΣΣ_*i*_*I*_*h*_, where *I*_*h*_ is the mean intensity for reflection *h*.

^c^*R*_pim_ = Σ_*h*_ (1/*n*_*h*_ − 1) Σ_*l*_ |*I*_*hl*_ − (*I*_*h*_)|/Σ_*h*_Σ_*l*_(*I*_*h*_).

^d^Correlation coefficient between random half datasets.

^e^*R*_cryst_ = Σ‖*F*_*o*_| − |*F*_*c*_‖/Σ|*F*_*o*_|, where *F*_*o*_ and *F*_*c*_ are measured and calculated structure factors, respectively.

^f^*R*_free_ = Σ‖*F*_*o*_| − |*F*_*c*_|/Σ|*F*_*o*_|, calculated from 5% of the reflections selected randomly and omitted during refinement.

**Figure 1:**
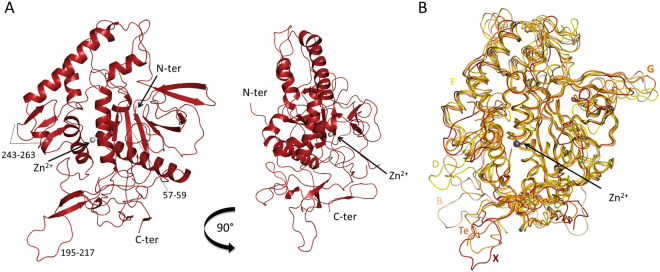
Crystal structure of LC/X. (**A**) Ribbon representation of LC/X, the zinc ion (grey sphere) and other regions of interest are highlighted. (**B**) Comparison of LC/X with other VAMP-cleaving toxins. LC of serotypes B (sand, PDB 1EPW), D (yellow-green, PDB 2FPQ), F (gold, PDB 2A97), G (dark orange, PDB 1ZB7) and Te (light orange, PDB 1Z7H) were superimposed with LC/X (red).

Despite only ~30% identity at the amino acid levels with other BoNTs, the overall structures of LC/X present a fold similar to other BoNTs with largely conserved secondary structural elements (Fig. 1). This high degree of similarity between LC/X and other BoNTs are reflected by the low root mean square deviations (rmsd) values between 2.3 and 2.9 Å when compared to other BoNT-LCs (Fig. 1B, Table 2, Supplementary Fig. S1). Interestingly, two of the BoNT-LCs that showed the highest sequence identity to LC/X (LC/B, 36%; LC/Te, 34%, for the structurally aligned residues) showed the least structural resemblance (rmsd of 2.8 Å and 2.9 Å, respectively). These differences between the structures rest mainly on the higher variations in the length and position of the flexible loop regions (Fig. 1C, Supplementary Fig. S1). LC/D, which also cleaves VAMP1/2/3, is the closest structural homologue (rmsd = 2.3 Å; *Z*-score = 47.1). However there was no general correlation between structural similarity and substrate preference, contrary to what was observed in the primary sequence pairwise alignments where the SNAP-cleaving BoNT have the lowest similarity to LC/X (Table 2). These data demonstrate that LC/X is a bona fide member of BoNT-LCs.

**Table 2:** Pairwise comparison of LC/X with other clostridial neurotoxins using Dali^37^ and Needle (EMBOSS server).

	Dali						Needle		
	PDB	rmsd	No. of aligned positions	No. of residues in matched structure	% Sequence identity of aligned positions	*Z* score	% Sequence similarity	% Sequence identity	Score
LC/D	2fpq	2.3	377	414	31	47.1	48	28	500
LC/F	2a97	2.3	365	392	33	46.4	47	30	520
LC/C	2qn0	2.3	384	429	31	45.8	47	31	507
LC/A	1xtf	2.4	389	427	32	45.4	44	29	465
LC/G	1zb7	2.4	367	414	36	44.0	49	33	546
LC/E	1t3a	2.5	370	400	30	47.0	45	29	434
LC/B	1f82	2.8	381	424	36	45.7	48	33	590
LC/Te	1z7h	2.9	384	426	34	46.5	48	31	549

**Figure 2:**
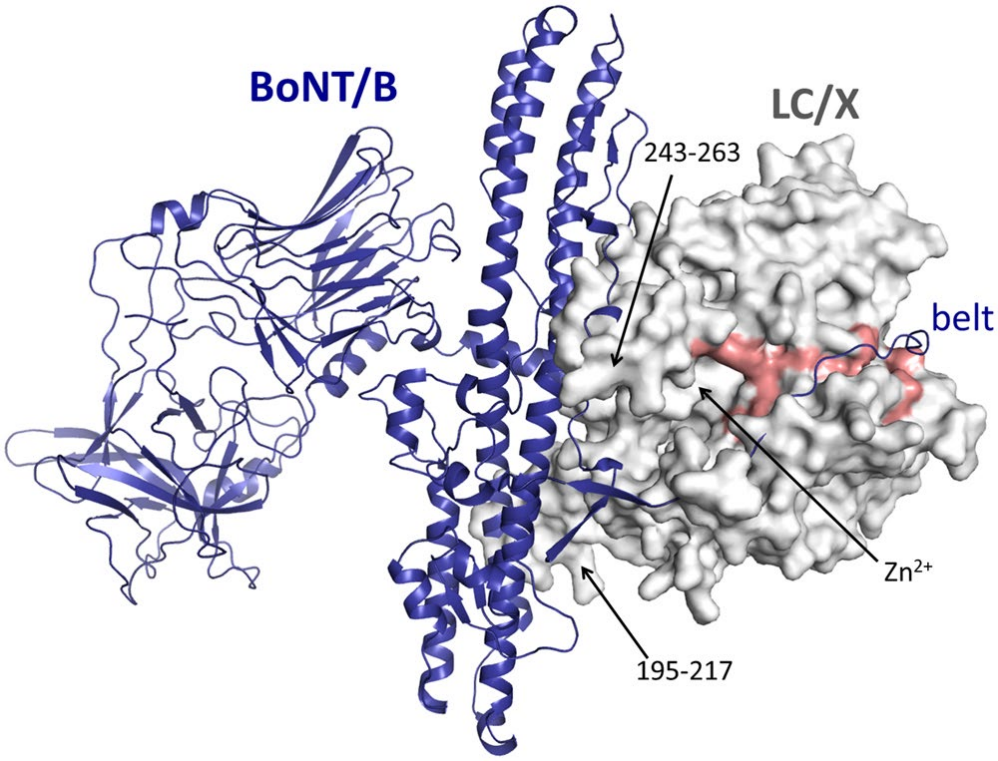
Superposition of LC/X with BoNT/B. LC/X was superposed to the LC of BoNT/B (PDB code 1EPW^16^) to illustrate the potential interaction with the translocation domain. LC/X is shown as a white surface while BoNT/B is showed as a blue ribbon (with its LC omitted from the structure for clarity). Residues flanking the groove of LC/X are highlighted in red.

Notable structural differences between LC/X and other BoNT-LCs include the loop region 195–217 (Fig. 1A). It starts and ends with anti-parallel β-strands, which are longer than the corresponding regions in LC/B and orientate the rest of the unstructured loop towards the solvent accessible surface. While the majority of the LC fold usually remains identical no matter whether the LC is an isolated protein or within the context of the full-length toxin (holotoxin), similar loop regions in other BoNT-LCs have showed flexibility to accommodate interactions with BoNT-HC^3,18^, suggesting that the loop 195–217 might contribute to inter-domain interactions within BoNT/X. Indeed, superposition of LC/X with BoNT/B hints that loop 195–217 may interact with the central helices of the translocation domain (Fig. 2).

In addition to loop 195–217, a major difference was observed for one of the loops involved in SNAP-25 binding by LC/A known as loop-250^7^, which in LC/X makes a three-stranded β-sheet arrangement from residues 243 to 263 and extends away from the catalytic channel, in a structural feature unique to LC/X. Loop-250 was shown to take on a different conformation in LC/A where it went from an open configuration in its free form to a closed configuration upon substrate binding^7^. Interestingly this loop corresponds to a random coil in other VAMP-cleaving LCs, with the exception of LC/F where it makes a β-hairpin, all have a length of 16 to 20 amino acids (Supplementary Fig. S2). Superposition of LC/X with BoNT/B suggests that region 243–263 could interact with the translocation domain in full length BoNT/X (Fig. 2), in agreement with this loop’s apparent flexibility.

### Active site of LC/X and the structure of zinc-free LC/X

**Figure 3:**
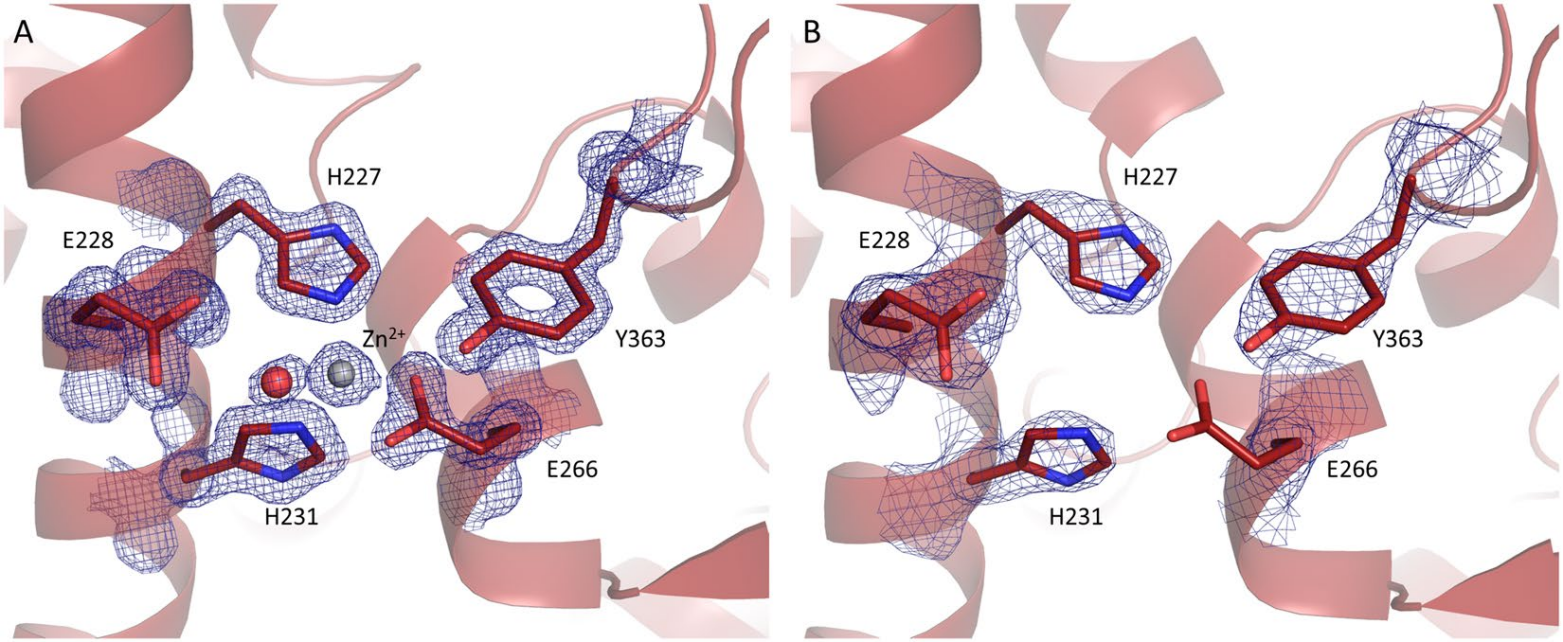
Catalytic site of LC/X. Close-up view of the active site of LC/X in (**A**) the high-resolution structure of LC/X; and (**B**) the Apo-LC/X structure. Residues involved in proteolysis represented in sticks, zinc ion and water molecules as grey and red spheres, respectively. The electron density (2*F*_*O*_*-F*_*C*_, contoured at 2σ in both (A) and (B)) is shown as a blue mesh.

BoNTs are metallo-endopeptidases of the M27 family^19^ related to thermolysin, with a classical HExxH+E tetrahedral zinc-binding motif. The active site of LC/X has the overall conserved geometry shared with other BoNTs (Supplementary Fig. S3). The Zn^2+^ ion is coordinated by the triad His227, Glu228 and His 231 and a fourth coordination provided by a water molecule linked to Glu266 (Fig. 3A). The water molecule provides the nucleophile base required for the proteolytic activity. Additionally, the conserved Tyr363 is believed to stabilize catalytic intermediates^15^ and is within water-mediated hydrogen bond distance to the active site.

In the second crystal form, which lacks Zn^2+^, the architecture of the active site did not present any rearrangement, demonstrating that the role of zinc is functional rather than structural (Fig. 3B). Only weak electron density could be observed for Glu266, which may be an indication of the side chain motility in absence of Zn^2+^ coordination. Additionally, residues 411–421 present a unique beta hairpin configuration in the apo-LC/X structure. This region could not be modelled in the high-resolution structure due to weak electron density, which suggests that the LC/X’s carboxy-terminal end is flexible.

### Access to the active site of LC/X is more restricted

**Figure 4:**
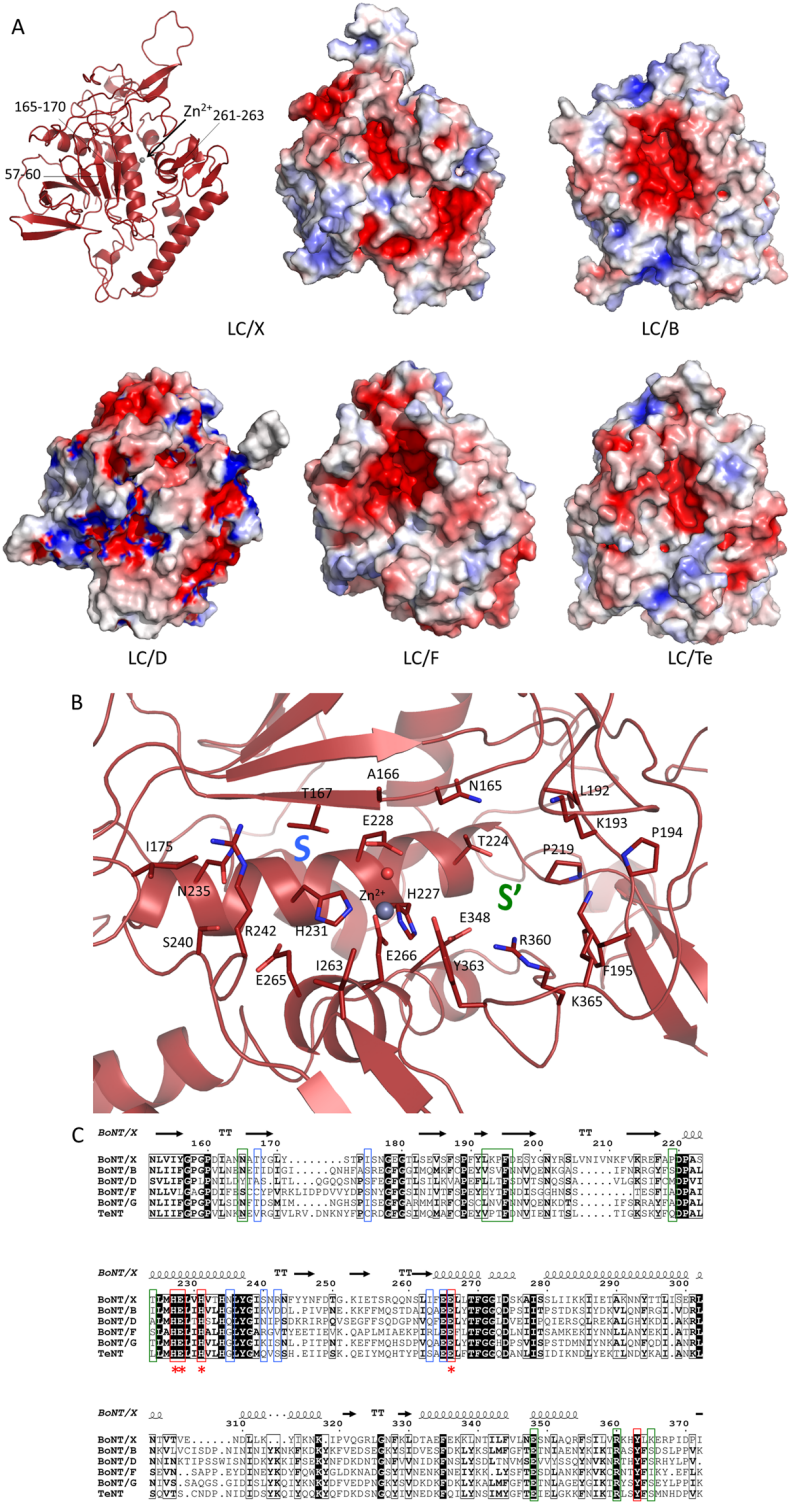
Surface potential and catalytic cleft of LC/X. (**A**) Left, ribbon representation of LC/X; right, surface representation of LC/X, /B, /D, /F and /Te with electrostatic potential calculated using the APBS tool in PyMol. (**B**) The substrate binding sites of LC/X highlighting the *S* an *S’* sub-pockets. (**C**) Sequence alignment of LC/X with other VAMP-cleaving toxins. Catalytic site residues are framed in red, residues of the *S* an *S’* sub-pockets are framed in blue and green, respectively. Stars denote amino acids involved in Zn^2+^ coordination.

Analysis of the surface accessible electrostatic potential highlighted the conserved negative electrostatic potential of the active site, deep inside an open cavity (Fig. 4). Interestingly, access to the catalytic pocket of LC/X appeared more restricted than in LC/B, and /F (Fig. 4). LC/D, the closest structural homologue of LC/X also presents a restrictive access to the catalytic site but contrast by its significantly more charged surface potential. The catalytic pocket of LC/X shows a narrow entrance flanked on one side by loop 57–60, composed of polar residues (Thr-Asn-Asn-Thr), and on the other side by the short 261–263 β-strand (Fig. 4A,B). Deeper within the cleft, residues 160–165 form a β-strand that borders the catalytic channel, with the *S* sub-pocket delineated by residues 235–242 (Fig. 4B). The *S* sub-pocket of LC/X is not particularly conserved compared to the other VAMP-cleaving toxins (Fig. 4C). These structural elements may be involved in substrate recognition and allow LC/X to cleave VAMPs at a specific and unique position^20^. On the other end, the *S’* sub-pocket delimited by loops 192–195 and 360–365 is well conserved (Fig. 4C), and may help stabilise the interaction with the C-terminal end of the substrate upon binding. In particular, Arg360, which is strictly conserved across all clostridial neurotoxins, likely plays a key role in substrate interaction, similarly to its role described in BoNT/A^21^. Consistently, mutations of Arg360 and Tyr363 in BoNT/X abrogated the toxin’s activity^17^.

### Comparison to LC/F-VAMP structure

**Figure 5:**
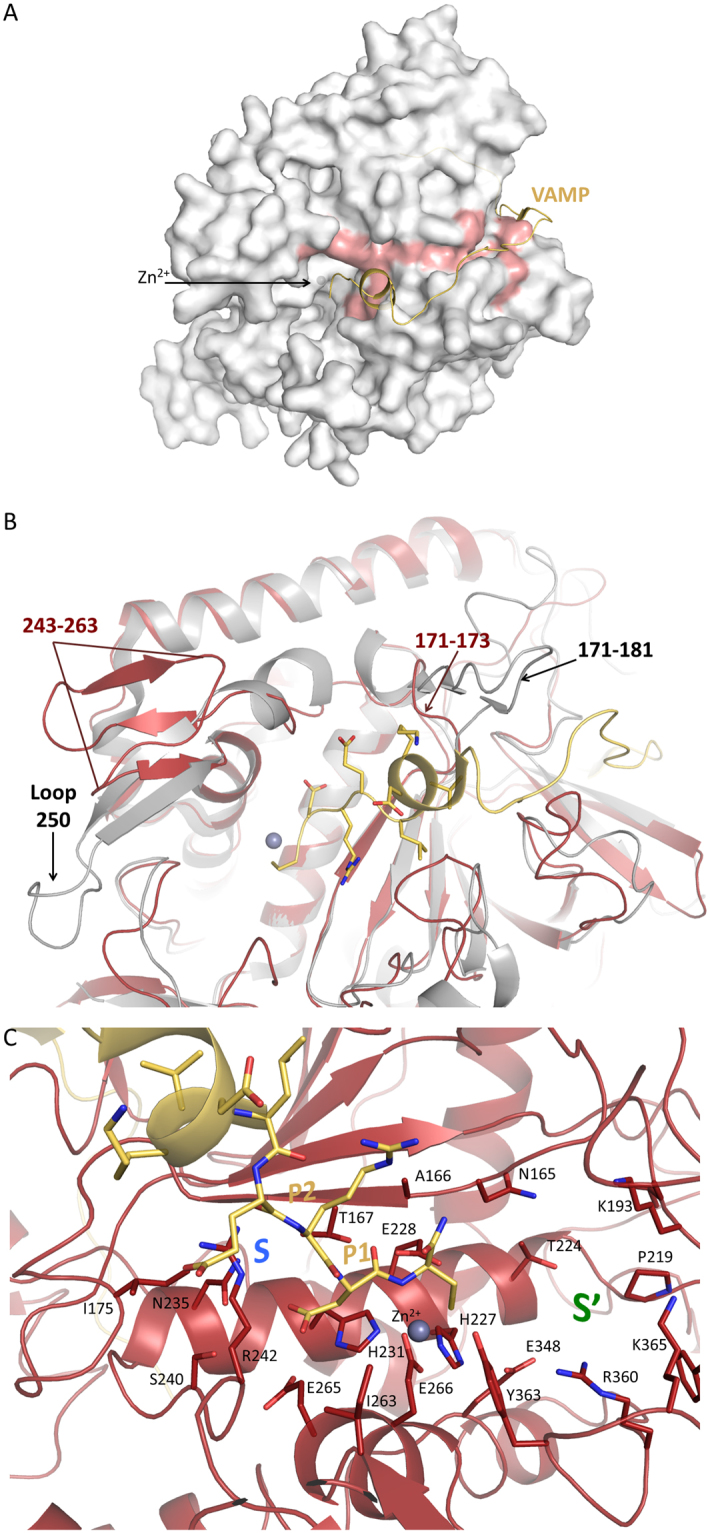
Comparison of LC/X to LC/F in complex with a VAMP-derived peptide inhibitor. LC/F in complex with a VAMP-derived inhibitor (PDB 3FIE) was superposed to LC/X. **(A)** The surface of LC/X is shown in white and the VAMP peptide is represented as a yellow ribbon (LC/F is not shown). Residues flanking the groove and potential exosites are highlighted in red. **(B)** Close-up view of the catalytic pocket highlighting the variations in the surrounding loops and exosites. LC/F is shown as a grey ribbon. **(C)** Close-up view of LC/X catalytic pocket and potential substrate binding sites. The bound VAMP inhibitor is shown as sticks (LC/F is not shown) and residues of LC/X potentially involved in substrate binding are presented as sticks.

Superposition of LC/X with the structure of LC/F bound to a VAMP-based peptide inhibitor^8^ revealed a clear groove that extends from the catalytic site and all around LC/X (Fig. 5A). This structural feature is common to other BoNTs and is expected to be involved in extended substrate interactions. In addition, superposition of LC/X with the full length BoNT/B (Fig. 2) suggests that this groove also likely interacts with the so-called ‘belt’ region of BoNT/X. The belt region surrounds the light chain in the full-length toxins and was shown to play a protective chaperone role in BoNT/A^22^.

The comparison with the VAMP-bound LC/F also highlighted a significant difference at the loop defining exosites 1 and 2 in LC/F (residues 171–181)^8^, which corresponds to a much shorter three-residue long linker (171–173) in LC/X (Fig. 5B). This difference in exosites may explain their distinct cleavage sites on VAMP2. Assuming that VAMP binds to LC/X in the canonical direction, the LC/F peptide inhibitor is expected to fill the *S* subsite following a pattern where the C-terminal cysteine interacts with Zn^2+^. Based on this assumption, we further analysed the catalytic pocket to understand the variation in substrate specificity (Fig. 5C).

An unusual feature of LC/X is its tolerance for residue changes at the cleavage site: it cleaves VAMP1/2/3 between R-A, VAMP4 and Ykt6 between K-S, and VAMP5 between R-A (Supplementary Fig. S4). We note that the residues at the P1 site (R and K) are positively charged. It is possible R/K may interact with the negatively charged or polar side chains of residues E265, S240 and N235 located within the S1 site. On the other side of the scissile bond, the P1’ site (A or S) may be accommodated between Tyr363 and Asn165. At the P2’ site, the negatively charged side chain of E or D may be involved in salt bridge formation with Arg350 further within the *S’* sub-pocket. Deeper within the catalytic cleft, positions P3’-P4’ (A/V and L) of VAMP1/2/3 and Ykt6 are likely to make hydrophobic interactions towards Pro194, Phe195 and Pro219. We note that the P3’ position in VAMP4/5 consists of polar residues (S and Q) that may come within hydrogen bonding distance of Lys193 and Lys365.

### LC/X has a higher cleavage efficiency than LC/B and LC/Te

As the substantial differences in the accessibility to the active site in LC/X may influence the efficiency of substrate cleavage, we next compared the cleavage kinetics between LC/X versus LC/B and LC/Te using a fluorescence resonance energy transfer (FRET) based toxin sensor. This sensor was based on the toxin reporter previously developed^23^, but replacing the original cyan fluorescent protein (CFP) and yellow fluorescent protein (YFP) with a new generation of fluorescent proteins, mRuby2 and Clover, as the FRET pair. We also replaced the linker between the two fluorescent proteins from the original VAMP2 to VAMP1, as VAMP1 is the dominant form expressed at peripheral motor nerve terminals^24^. Cleavage of VAMP1 by toxins separates mRuby2 and Clover, thus reducing the FRET signal.

**Figure 6:**
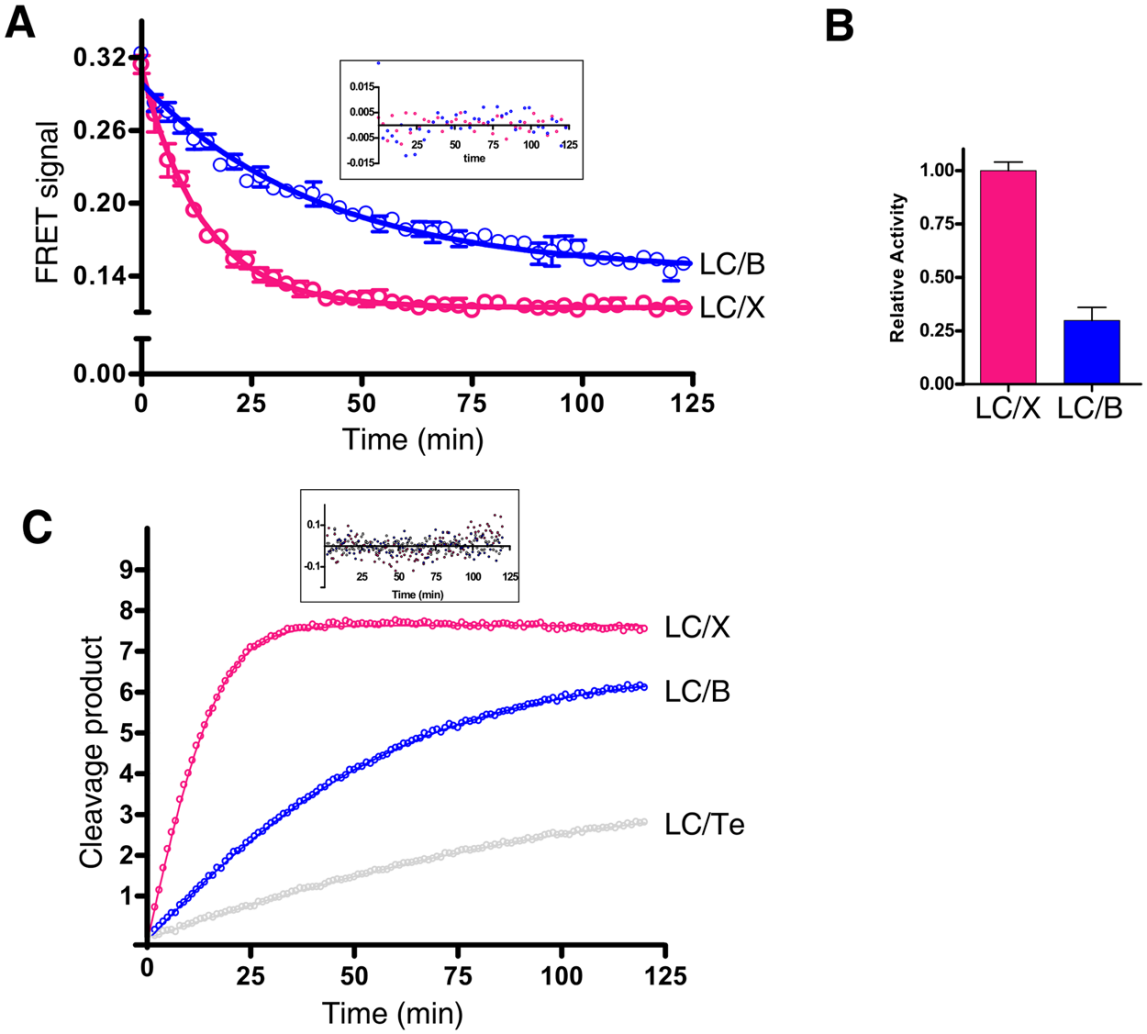
LC/X showed a higher level of cleavage efficiency than LC/B and /Te. **(A)** FRET-based toxin sensor (500 nM) was incubated with LC/X or LX/B (20 nM each). FRET signals were recorded with a microplate reader. Traces represent triplicates of experiments. The data were fitted to a single exponential function (Error bars are SEM; inset are the residuals of the exponential fit). The fitting data were presented in Supplementary Table S1. **(B)** Relative activity of LC/B was normalized to the activity of LC/X based on their rate constants estimated from panel A. **(C)** FRET-based toxin sensor (7.5 µM) was incubated with LC/X (2.4 nM), LC/B (4.8 nM) or LC/Te (92 nM). The cleavage products (Y-axis, µM) were estimated based on normalizing the FRET signal to the initial signal with 7.5 µM toxin sensor. The data were fitted to a 3-parameter equation to estimate the kinetic constants for each enzyme (inset: residuals of fit). The kinetics data were presented in Table 3.

As shown in Fig. 6A, incubation of the FRET sensor (500 nM) with LC/X or /B (20 nM each) resulted in loss of FRET signal over time. The traces were fitted to a one-phase single exponential decay and the rate constants were determined as a measurement for activity. LC/X showed ~3–4 fold more activity than LC/B towards the VAMP-1 substrate, with a reaction rate of 0.07 and 0.02 min^−1^ for LC/X and LC/B, respectively (Fig. 6B, Table 2).

We next estimated cleavage kinetics by using lower levels of LCs. To generate a time-dependent enzyme progress curve, the sensor concentration was increased to 7.5 µM and enzyme concentrations were optimized based on estimated relative activities (2.4 nM LC/X, 4.8 nM LC/B, 92 nM LC/T). The cleavage product concentration was estimated based on normalizing the FRET signals to the signal at the initial sensor concentration (7.5 µM, Fig. 6C). The data were then fitted to estimate kinetic parameters, which are listed in Table 3. The estimated kinetic parameters for LC/B and LC/Te are similar to previously reported values^25^. The kinetic data confirmed that LC/X is substantially more efficient than LC/B (~10 fold) and LC/Te (~30 fold). This enhanced cleavage efficiency is largely due to a higher apparent turnover rate (270 min^−1^) of LC/X towards VAMP1 than LC/B and LC/Te.

**Table 3:** Lambert-Omega Function parameters (see Fig. 6C).

	V (μM/min)	Km (μM)	Apparent rate (min^−1^)	Apparent efficiency (min^−1^/μM)
LC/X	0.65 (0.03)	4.3 (0.3)	271	63
LC/B	0.22 (0.01)	8.2 (0.8)	46	6
LC/Te	0.06 (0.01)	2.8 (0.8)	7	3

In summary, the structural characteristics of LC/X highlighted several unique features such as the restricted access to the catalytic pocket and its surrounding loop regions. The location and composition of the potential VAMP-binding pockets and the groove surrounding LC/X have implications with regard to substrate binding and interaction of LC/X with the rest of the holotoxin. Key regions of the light chain likely exhibit a high level of flexibility to adapt from the tight interactions with the belt and translocation domain of BoNT/X, to a different conformation upon substrate recognition when the free LC reaches its intracellular neuronal target. Our studies also revealed that LC/X possesses > 10-fold higher level of cleavage efficiency than LC/B and /Te, potentially due to the distinct features surrounding its active site. Future work on mutagenesis approaches and structural studies of LC/X in complex with VAMPs are required to pinpoint the exosites and fully understand substrate recognition. LC/X showed a high degree of tolerance for residues at the cleavage site and it has a higher level of activity than many BoNTs, it may serve as a promising candidate to engineer new toxin variants that can cleave SNARE proteins that currently cannot be targeted, such as VAMP7 and VAMP8, to provide novel scientific tools and therapeutic toxins.

## Methods

### LC/X expression and purification

The gene for LC/X was synthesized and cloned into a pET/28a vector for expression in *E*.*coli* BL21 (DE3). Cultures were grown at 37 °C and induction carried out at 18 °C using 0.5 mM IPTG. Bacterial pellets were lysed with an Emulsiflex-C3 (Avestin, Germany) at 20 kPsi and the protein purified by affinity chromatography using a 5 ml HisTrap HP column (GE Healthcare) followed by gel filtration with a 16/60 Superdex 200 column (GE Healthcare). The final sample was stored at 13.6 mg/ml in 20 mM HEPES pH7.5, 300 mM NaCl, 10% Glycerol and 2 mM TCEP.

### LC/X crystallisation and structure determination

Crystals of LC/X were obtained in two different forms. One presented prism-shaped crystals that grew within 24 h at 21 °C using the hanging drop vapour-diffusion method where 1 μl of protein at 13.6 mg/ml was mixed with 1 μl of reservoir solution consisting of 19% v/v PEG1500, 0.1 M MMT pH 6.0. The second form presented plate-like crystals that grew within 2–3 days at 21 °C with a similar set up and a reservoir solution consisting of 11% v/v PEG20000, 0.1 M MES pH 6.5, 10 mM EDTA. The crystals were transferred into a cryo-protectant solution consisting of their respective growing conditions supplemented with 25% v/v glycerol, and then frozen in liquid nitrogen for data collection. Diffraction data were collected at stations I02 of the Diamond Light Source (Didcot, UK), equipped with a PILATUS-6M detector (Dectris, Switzerland), and PXI of the Swiss Light Source (Villigen, Switzerland) equipped with a EIGER-16MX detector (Dectris, Switzerland). Complete datasets to 1.35 Å and 2.4 Å were collected from single crystals at 100 K. Raw data images were processed and scaled with Mosflm^26^, or XDS^27^ and AIMLESS^28^ using the CCP4 suite 7.0^29^. The structure was solved using molecular replacement with the coordinates of a truncated LC/B (PDB code 1EPW^16^) as a search model in PHASER^30^. The working models were refined using REFMAC5^31^ and manually adjusted with COOT^32^. Water molecules were added with the help of ARP/wARP^33^ at positions where *Fo−Fc* electron density peaks exceeded 3σ, and potential hydrogen bonds could be made. Validation was performed with MOLPROBITY^34^. Crystallographic data statistics are summarized in Table 1. All figures were drawn with PyMOL (Schrödinger, LLC, New York).

### FRET-based toxin sensor

The FRET toxin sensor was constructed by fusing the Clover and mRuby2 fluorescent proteins to each ends of a human VAMP1 fragment (residues 34–87, GenBank No. CAA88760.1), and was cloned into pBAD/His-B vector using Gibson assembly. Electrocompetent *E*. *coli* strain DH10B was transformed with the plasmid. Transformed bacteria were cultured overnight on agar plates with 0.4 mg/ml ampicillin. A single colony was picked and grown overnight in 5 mL LB medium supplemented with 0.1 mg/ml ampicillin at 37 °C. The 5 mL culture was then used to inoculate 500 mL of LB medium with ampicillin and grown to an optical density of 0.6. Protein expression was induced with the addition of 0.02% w/v arabinose and the culture was grown at 37 °C for 4 h and then 20 °C overnight. Bacteria were than harvested and the FRET sensor protein was purified from the supernatant by Ni-NTA affinity chromatography. The buffer of the purified protein was then exchanged to 10 mM Tris-HCl, 150 mM NaCl, pH 7.2 using a desalting column.

### Cleavage kinetics analysis

The cleavage assay was performed in a 50 µL volume at 37 °C, with 500 nM FRET sensor in the assay buffer (50 mM HEPES, pH 7.1, 200 mM NaCl, 10 µM ZnCl_2_, 500 µg/mL BSA, 2 mM DTT). The FRET signals were detected using microplate reader in 96-well plates at 620/520 nm. In Fig. 6A, the data were fit to a one-phase single exponential decay using GraphPad Prism 4 software. In Fig. 6C, FRET sensor concentration was increased to 7.5 µM. The LC/X concentration was optimized experimentally and we selected 2.4 nM, which is sufficient to turn over all substrates within the assay time (~ 30 min). Higher concentrations of LC/B (4.8 nM) and LC/T (92 nM) were used as these LCs showed lower activity compared to LC/X. Data were converted to a product accumulation plot by first transforming Y-axis (dividing 1 over the FRET signal), subtracting the baseline (the value from the control sample containing LC/X, FRET sensor, and EDTA), and then the concentration was deducted by normalizing to the value of the initial substrate concentration (7.5 µM). The data were then fitted to equation (1) to estimate kinetic parameters^35,36^.

1 $$Y=W(x(t))=W\{\frac{[S]\circ }{KM}\cdot exp(\frac{[S]\circ -V\cdot t}{KM})\}$$

where W is the Lambert W(x) function approximation (2):

2 $${W}^{w}(x)=ln\,(1+x)\cdot \{1-\frac{ln\,(1+ln\,(1+x))}{2\,+\,ln\,(1+x)}\}$$

All analysis and fitting were performed in Synergy KaleidaGraph 4 or GraphPad Prism 4.

### Data availability

The atomic coordinates and structure factors (codes 6F47 and 6F4E) have been deposited in the Protein Data Bank (http://wwpdb.org).

## Electronic supplementary material

Supplementary Information
