# Supplementary material for: Structural characterisation of the catalytic domain of botulinum neurotoxin X - high activity and unique substrate specificity
Source: Sci Rep. 2018 Mar 14;8:4518. doi: 10.1038/s41598-018-22842-4 (PMC5851995; doi:10.1038/s41598-018-22842-4)
Supplement: Supplementary file 1 — Supplementary Information [file 41598_2018_22842_MOESM1_ESM.pdf]

Structural characterisation of the catalytic domain of botulinum neurotoxin X - high activity and unique substrate specificity

Geoffrey Masuyer, Sicai Zhang, Sulyman Barkho, Yi Shen, Linda Henriksson, Sara Kosenina, Min Dong and Pål Stenmark

Supplementary information:

Supplementary Figure S1: Sequence alignment of LC/X with other VAMP-cleaving LCs

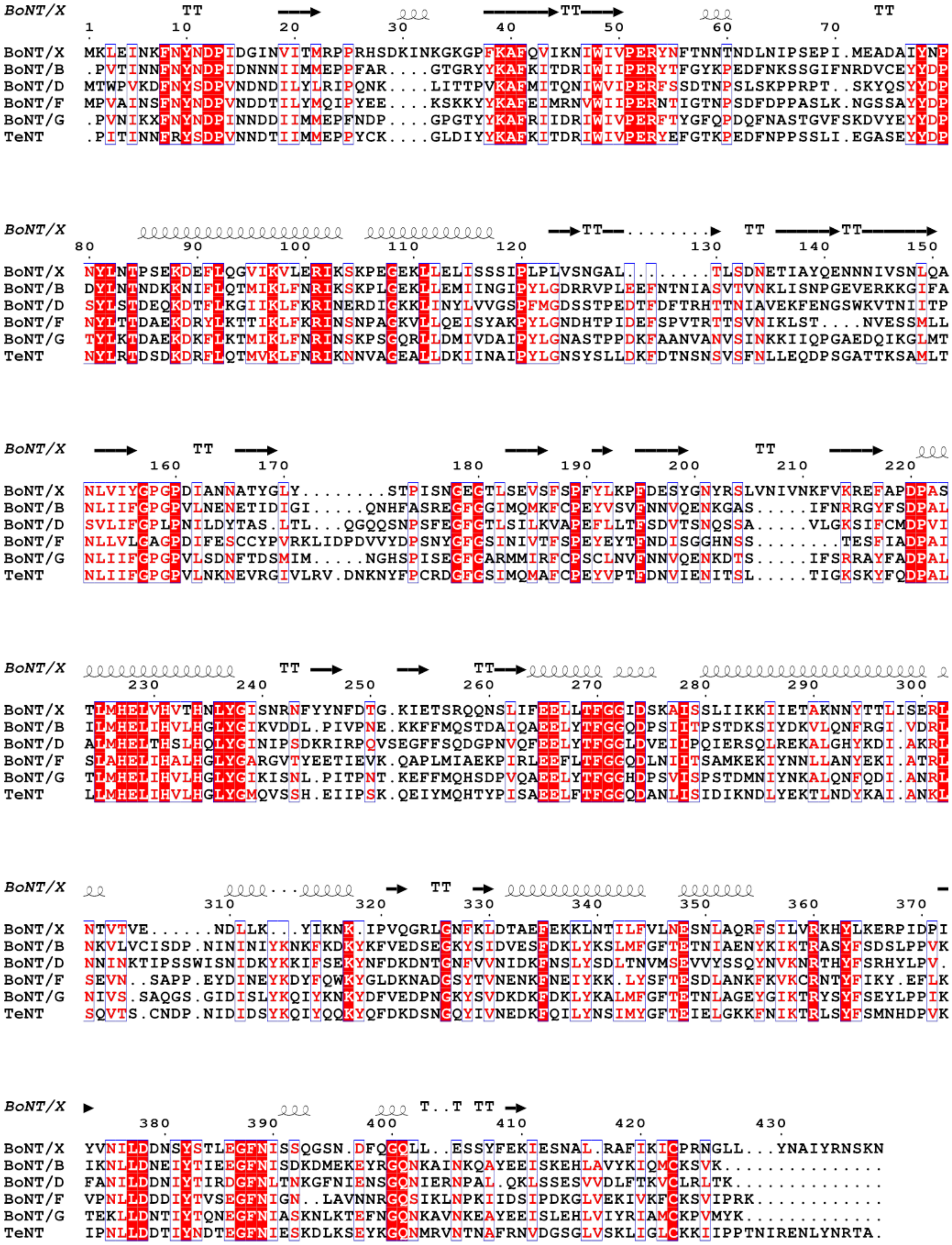

Supplementary Figure S2: Loop 250: Comparison of the loop between LCs

Ribbon representation of LC/X (Red), compared to the LC of serotypes A, (cyan, PDB 3BON), B (sand, PDB 1EPW), D (yellow, PDB 2FPQ), F (gold, PDB 2A97), and Te (light orange, PDB 1Z7H). The zinc ion is shown as a grey sphere.

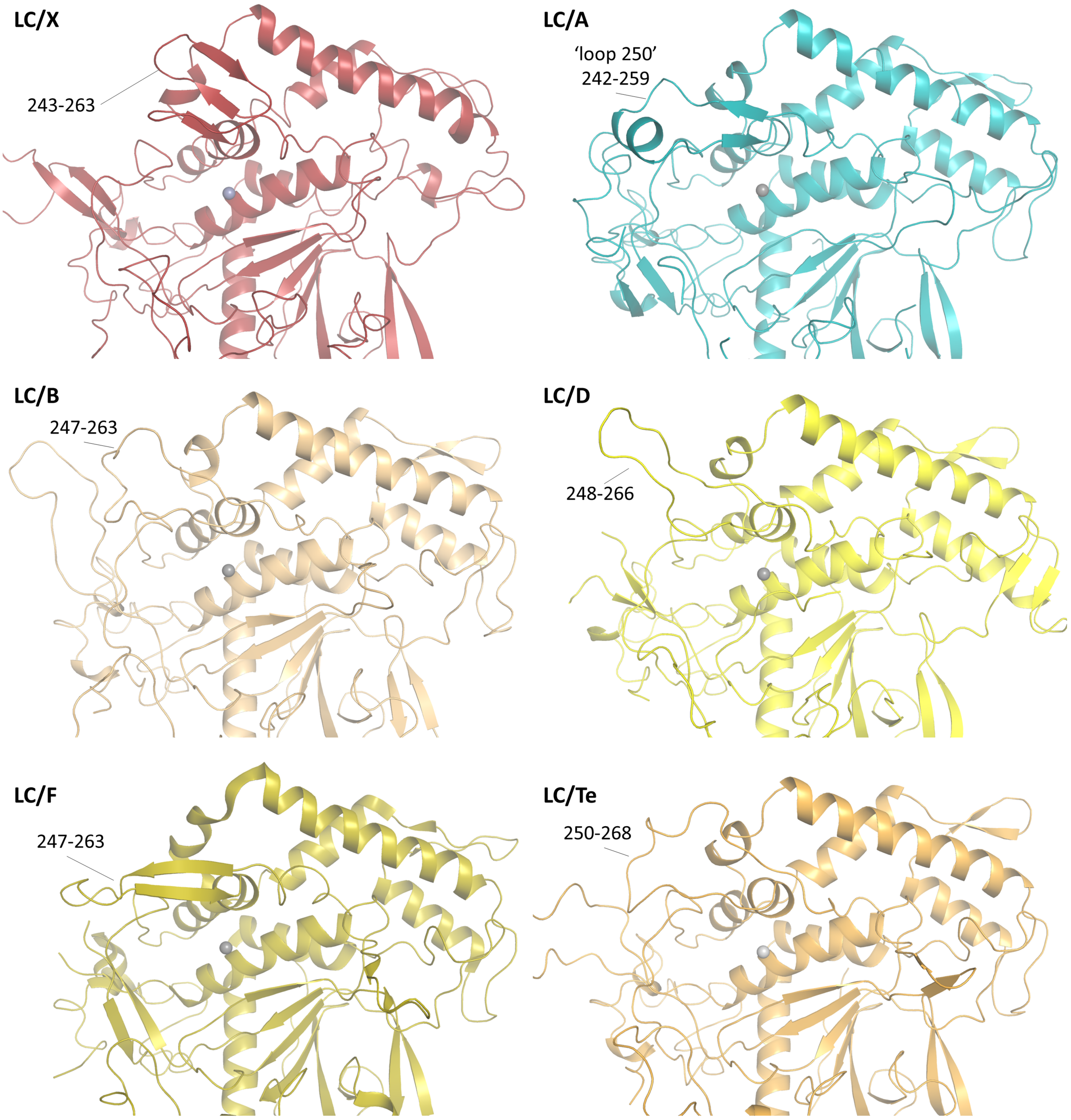

Supplementary Figure S3: Catalytic site: conserved Zn^{2+} coordination

LC/X (Red) was superposed with the LC of serotypes A, (cyan, PDB 3BON), B (sand, PDB 1EPW), D (yellow, PDB 2FPQ), F (gold, PDB 2A97), and Te (light orange, PDB 1Z7H). The zinc ion is shown as a grey sphere and the zinc-coordinating residues as sticks.

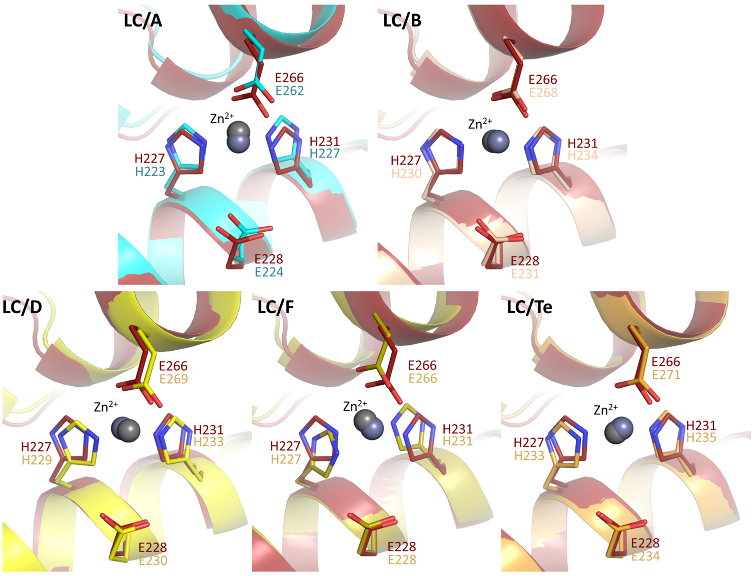

Supplementary Figure S4: LC/X cleavage sites on its VAMP substrates.
Cleavage site is highlighted in red.

```
VAMP1    35. QQTQAQVEEVVDIIRVNVDKVLERDQKLSELDDRADALQAGASQFESSAAKLKR .88
VAMP2    33. QQTQAQVDEVVDIMRVNVDKVLERDQKLSELDDRADALQAGASQFETSAAKLKR .86
VAMP3    20. QQTQNQVDEVVDIMRVNVDKVLERDQKLSELDDRADALQAGASQFETSAAKLKR .73
VAMP4    54. KHVQNQVDEVIDVMQENITKVIERGERLDELQDKSESLSDNATAFSNRSKQLRR .107
VAMP5     7. ERCQQQANEVTEIMRNNFGKVLERGVKLAELQQRSDQLLDMSSTFNKTTQNLAQ .60
Ykt6    140. TKVQAELDETKIILHNTMESLLERGEKLDDLVSKSEVLGTQSKAFYKTAR---- .189
```

Supplementary Table S1: Exponential fit parameters (see Fig. 6A)

	**A**	**k (min^{-1})**	**C**	**R^2**
**LC/X**	0.19	0.07	0.11	0.95
**LC/B**	0.15	0.02	0.14	0.94
